# Collagen and microvascular alterations contribute to neuromuscular degeneration and disease progression in chronic intestinal pseudo‐obstruction

**DOI:** 10.1111/joim.70078

**Published:** 2026-02-27

**Authors:** Elisa Boschetti, Irene Neri, Leonardo Caporali, Elena Bonora, Carolina Malagelada, Claudio Fiorini, Danara Ormanbekova, Alessandro Berghella, Roberto D'Angelo, Rita Rinaldi, Cristiana Caliceti, Anna Costanzini, Mirella Falconi, Vincenzo Stanghellini, Stefano Ratti, Lucia Manzoli, Valerio Carelli, Roberto De Giorgio

**Affiliations:** ^1^ Cellular Signalling Laboratory, Anatomy Center, Department of Biomedical and Neuromotor Sciences (DIBINEM) University of Bologna Bologna Italy; ^2^ IRCCS Istituto delle Scienze Neurologiche di Bologna, Programma di Neurogenetica Bologna Italy; ^3^ IRCCS Azienda Ospedaliero‐Universitaria di Bologna Bologna Italy; ^4^ Department of Medical and Surgical Sciences (DIMEC) University of Bologna Bologna Italy; ^5^ Digestive System Research Unit Vall d'Hebron University Hospital Barcelona Spain; ^6^ Centro de Investigación Biomédica en Red de Enfermedades Hepáticas y Digestivas (Ciberehd) Barcelona Spain; ^7^ Neurogenetics Laboratory, Department of Biomedical and Neuromotor Sciences (DIBINEM) University of Bologna Bologna Italy; ^8^ IRCCS Istituto delle Scienze Neurologiche di Bologna, UO Neurologia AOU Bologna Italy; ^9^ Laboratory of Precision Biochemistry of Longevity and Age‐Related Pathologies, Department of Biomedical and Neuromotor Sciences (DIBINEM) University of Bologna Bologna Italy; ^10^ Department of Translational Medicine University of Ferrara Ferrara Italy

**Keywords:** chronic intestinal pseudo‐obstruction, fibrosis, intestinal dysmotility, small vessels

## Abstract

**Background:**

Chronic intestinal pseudo‐obstruction (CIPO) is a severe gastrointestinal motility disorder that may be idiopathic or associated with systemic disease. In idiopathic cases, the pathophysiological mechanisms remain poorly defined. Although mutations in angiogenic factors have been reported in mitochondrial forms of CIPO, their role in non‐mitochondrial cases is still unclear.

**Objective:**

To investigate genetic and molecular contributors to CIPO, with a specific focus on intestinal microvasculature.

**Methods:**

Jejunal samples from patients with CIPO were analysed by whole exome sequencing (WES) and mitochondrial DNA (mtDNA) profiling. Morphometric and immunohistochemical studies assessed collagen remodelling, vascular architecture, neuromuscular integrity and hypoxia. Expression of angiogenic factors, including thymidine phosphorylase (TP) and vascular endothelial growth factor (VEGF), was evaluated.

**Results:**

WES did not identify known CIPO‐causing variants, but rare mutations in collagen‐related genes were detected in a subset of patients. Tissue analysis revealed higher fibrosis, vascular remodelling with a predominance of very small vessels, thinning of the longitudinal muscle and neuronal loss. TP and VEGF expression were significantly reduced, whereas hypoxia‐inducible factor‐1α (HIF‐1α) was markedly upregulated. mtDNA integrity and copy number were preserved, whereas haplogroup J was overrepresented. Multivariate analysis linked these alterations to a higher frequency of sub‐occlusive episodes.

**Conclusions:**

Vascular dysfunction and collagen abnormalities emerge as key contributors to neuromuscular degeneration in CIPO. These findings provide novel mechanistic insights into disease pathophysiology and support further exploration of vascular‐targeted therapeutic strategies.

## Graphical abstract

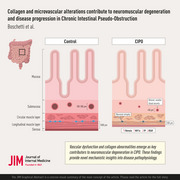

## Introduction

Chronic intestinal pseudo‐obstruction (CIPO) is a rare form of severe dysmotility characterized by radiologically proven recurrent sub‐obstructive episodes, often with a dilated intestine, in the absence of any lumen‐occluding lesion [1, 2, 3]. The common denominator of this condition is a marked impairment of gut propulsion due to abnormalities affecting key regulatory cells, including enteric neurons and glia, interstitial cells of Cajal (ICC) and smooth muscle cells [4, 5]. However, the underlying etiopathogenetic mechanisms affecting neuro‐ICC‐muscular integrity [6], thereby leading to severe gut dysmotility of CIPO patients, have not been elucidated yet.

CIPO can be secondary to a variety of metabolic, neurological, paraneoplastic and autoimmune disorders, but several cases are classified as idiopathic [7]. Genetic factors are increasingly recognized in patients with CIPO, with 15 causative genes identified to date [8]. However, these genetic mutations cannot be detected in most patients, which are thus diagnosed without a defined genetic cause. In this study, we first performed whole exome sequencing (WES) and screened mitochondrial DNA (mtDNA) in a cohort of clinically well‐characterized patients with CIPO in order to investigate the potential underlying genetic landscape. We then explored the hypothesis that CIPO may represent a convergent phenotype arising from the interplay of multiple pathogenic mechanisms.

Previously, we demonstrated that in CIPO associated with mitochondrial neurogastrointestinal encephalomyopathy (MNGIE), an ultra‐rare disease caused by *TYMP* gene mutations, the gastrointestinal (GI) tract exhibits extensive fibrosis, microvascular abnormalities, along with mitochondrial dysfunction [9, 10, 11]. CIPO‐MNGIE is characterized by a genetically determined deficiency of thymidine phosphorylase (TP) enzyme, reduced vascular density with poorly functional microvessels, neuromuscular atrophy, neuronal loss and a lower mtDNA copy number [9]. These findings established vascular and mitochondrial dysfunction as key pathogenic aspects underlying CIPO‐MNGIE [11].

To investigate whether similar pathogenic features may also contribute to non‐MNGIE CIPO patients, we performed a wide morphometric analysis on our cohort, assessing and quantifying tissue fibrosis, vascular network and neuromuscular integrity in full‐thickness jejunal biopsies. Based on the results of the morphometric analysis, we then focused on TP and vascular endothelial growth factor (VEGF), two pivotal angiogenic mediators in the GI tract, and on hypoxia inducible factor‐1α (HIF‐1α) as a marker of hypoxia. Finally, we evaluated mtDNA copy number to assess mitochondrial genome maintenance.

This integrated and translational approach was devised to explore novel insights into CIPO pathophysiology and uncover potential molecular targets for therapeutic intervention.

## Methods

### Patients and sampling

Full‐thickness jejunal samples were collected from  22 well‐characterized idiopathic CIPO patients (14 females, 8 males—sex assigned at birth; age range: 16–78 years) recruited at the Val d'Hebron Hospital (Barcelona, Spain) between November 1999 and November 2009. Full‐thickness intestinal biopsies are not required for the clinical diagnosis of CIPO. However, they may provide relevant pathophysiological information in carefully selected adult patients, that is, idiopathic cohorts, late‐onset, treatment‐refractory cases with inconclusive genetic testing and no identifiable secondary causes [5, 12]. Patients with motility disorders secondary to infectious, neurological, metabolic, systemic‐autoimmune and paraneoplastic conditions were excluded. Furthermore, cases with enteric dysmotility [4] or carrying previously known CIPO‐causative gene mutations were also excluded. Each CIPO diagnosis was confirmed by clinical and radiological (air‐fluid levels in dilated bowel loops) features mimicking mechanical obstruction of the gut [13]. Organic causes of mechanical obstruction of the gut were excluded by endoscopic and radiological examinations. Furthermore, small bowel manometry was performed in all patients as an adjunctive test to unravel possible neuromuscular abnormalities detectable in CIPO patients [14, 15]. Of note, opioid analgesics were not being administered to any of the enrolled patients at diagnosis or at the time of tissue sampling. Comparable jejunal samples used as control tissues were obtained from  10 (2 females, 8 males—sex assigned at birth; age range: 47–73 years) patients undergoing resection for non‐complicated GI tumours without infiltration and without any history of GI dysmotility or other GI disorders. As an additional precaution, control samples were collected from areas macroscopically distant from the tumour sites and were histologically examined to exclude subtle abnormalities such as fibrosis or inflammation. None of the control samples displayed such features. Tissue specimens from each subject were immediately formalin fixed in cold neutral 4% formaldehyde and paraffin embedded (FFPE). In a subset of patients and controls, the full‐thickness biopsy sample was divided into two pieces; one was snap frozen in liquid nitrogen and stored at −80°C for molecular analysis. The cohort used for this purpose (see Table S1) included 7/10 controls (age range: 47–73 years; 2 females) and 20/22 CIPO (age range: 16–78; 12 females). Clinical diagnosis information is detailed in Supporting Information section, whereas patients’ demographics and a summary of the signs and symptoms are reported in Table S1.

### Ethical statement

All the experimental procedures were carried out in accordance with the Declaration of Helsinki and were approved by the Ethics Committees of the Vall d'Hebron University Hospital and St. Orsola‐Malpighi Hospital for handling and analysis of tissue samples from patients with severe gut dysmotility (EM/146/2014/O). Informed consent was obtained from all the human subjects included in this study.

### WES and mtDNA sequencing analysis

Total DNA was extracted from 10 mg of starting tissue (full thickness), using the PureLink Genomic DNA Mini Kit (Invitrogen) [16, 17].

WES sample library was prepared from genomic DNA using xGen DNA EZ Library Prep Kit (Integrated DNA Technologies IDT) and enriched using xGen Exome Hyb Panel v2x with mtDNA Hyb Panel spike‐in (IDT). Sequencing was performed on a NovaSeq 6000 instrument (Illumina) with 100 bp paired‐end reads. Bioinformatic analysis followed GATK Best Practices workflow for germline variant discovery, aligning to reference genome GRCh37/hg19. We evaluated the variants pathogenicity according to the ACMG guidelines, particularly focusing on genes associated with GI neuromuscular disorders (Version 1.23, Green List, Genomics England PanelApp).

The sequence of mtDNA was analysed through the mtDNA‐Server 2 pipeline [18] that automatically assigns mitochondrial haplogroups according to PhyloTree—Build 17 (www.phylotree.org). We manually analysed non‐synonymous variants that did not define haplogroups, checking their frequency in GenBank and gnomAD databases [19] and the pathogenicity score prediction according to APOGEE 2. We also evaluated the presence of mtDNA deletions and duplications through the MitoSAlt pipeline [20].

The frequency of mtDNA haplogroups was calculated using only CIPO patients from our cohort with traceable Spanish origin (*n* = 18/20) and compared to a large cohort of healthy Spanish controls (*n* = 326) from a European haplogroup‐tracing database held at the IRCCS Institute of Neurological Sciences of Bologna. Logistic regression was corrected for multiple testing using the Benjamini–Hochberg method. To exclude potential confounding effects, univariate analysis was performed to assess the association between the primary outcomes and the following variables: age, disease duration, presence of bowel dilatation and nutritional status.

### Assessment of GI tissue

#### Fibrosis

The quantification of fibrosis was performed directly on FFPE tissue sections (10 µm) using the Sirius Red (that binds to all types of collagen)/Fast Green (for non‐collagenous proteins) staining kit (Condrex). Briefly,  2 non‐consecutive tissue sections for each subject were deparaffinized and incubated for 30 min with the Sirius red/fast green staining solution mix. After washing with distilled water, images were captured using an Evident/Olympus IX83 inverted microscope coupled with cellsens dimension software 4.3.1 at different magnifications for a qualitative assessment. Sirius red/fast green dye was extracted from each stained section using 1 mL of dye extraction buffer included in the kit. The eluted dye solution was collected in a plastic cuvette and analysed with a 350 UV–Vis spectrophotometer (Thermo Scientific) at *λ* = 540 nm (fast green) and *λ* = 605 nm (sirius red) to obtain the Optical Density (OD) value. Fibrosis was estimated using the ratio between collagenous and non‐collagenous protein and expressed as µg/section according to the following two formulas, respectively:

$$\begin{array}{c} \mathrm{Collagenous}\;\mathrm{protein}\;\left(\mu g/\mathrm{section}\right)=\;\frac{\left[\mathrm{OD}\;540\;\mathrm{value}-\left(\mathrm{OD}\;605\;\mathrm{value}\times 0.291\right)\right]}{\mathrm{colour}\;\mathrm{equivalence}\;\mathrm{on}\;\mathrm{non}-\mathrm{collagenous}\;\mathrm{protein}\;\mathrm{at}\;\mathrm{OD}\;540\left(=0.0378\right)} \end{array}$$

$$\begin{array}{c} \mathrm{Non}-\mathrm{collagenous}\mathrm{proteins}\left(\mu g/\mathrm{section}\right)=\;\frac{\mathrm{OD}\;605\;\mathrm{value}}{\mathrm{colour}\;\mathrm{equivalence}\mathrm{of}\mathrm{collagenous}\mathrm{protein}\mathrm{at}\mathrm{OD}\;605\left(=0.00204\right)} \end{array}$$

The average obtained from the control group has been used as a calibrator (unitary value). For an appropriate calculation of the collagen amount, the OD 540 value was corrected by subtracting the contribution of fast green at 540 nm, corresponding to 29.1% of the OD 605 value.

The fibrosis index was also evaluated after stratifying patients according to the presence of an inflammatory infiltrate in the neuromuscular layer. Patients were classified as ‘inflamed’ (INF; *n* = 8), including  6 patients with mast‐cell infiltrates and  2 patients with mixed mast‐cell and lymphocytic infiltrates in the neuromuscular layer, or as ‘non‐inflamed’ (NOT‐INF; *n* = 14), defined by the absence of inflammatory infiltrates. Specifically, patients were classified based on the presence of inflammatory cells in the neuromuscular layer. Lymphocyte infiltration was defined following the criteria of the International Working Group on GI neuromuscular pathology (≥1 intraganglionic and/or >5 periganglionic lymphocytes per ganglion) [21]. Mast cells were quantified using immunohistochemistry for the specific marker tryptase, and patients were considered to have an underlying inflammatory neuromyopathy if mast cells were detectable within the neuromuscular compartment as previously described [6, 22].

Statistical differences between CIPO and controls were calculated using a Mann–Whitney test (non‐parametric). Statistical significance was set at *p* < 0.05. Data are reported in the results section as per cent variation between CIPO patients and controls, with median and interquartile range (IQR) indicated in parentheses.

#### Circular and longitudinal muscle layer thickness evaluation

Sirius red/fast green stained sections were also used to measure the circular and the longitudinal muscle thickness using Imagej 1.48V software. For each patient and control, the final value has been obtained using the average of at least six measurements per section in random (non‐overlapping) fields containing the circular or the muscular longitudinal layer respectively. These measurements were performed on two separate nonconsecutive tissue sections by two investigators (blinded with regard to CIPO or control origin). Statistical differences between CIPO and controls were calculated using a Mann–Whitney test (non‐parametric). Statistical significance was set at *p* < 0.05. Data are reported in the results section as per cent variation between CIPO patients and controls, with median and IQR indicated in parentheses.

#### Myenteric and submucosal neuronal quantification and myenteric inter‐ganglionic distance assessment

We quantified the number of neurons per ganglion and measured the distance between myenteric ganglia using a previously published method [6]. The procedure is specified in Supporting Information. To calculate differences in myenteric and submucosal ganglia or inter‐ganglionic distance between CIPO patients and controls, a Mann–Whitney test (non‐parametric) was used for each analysis. Statistical significance was set at *p* < 0.05. Data are reported in the results section as per cent variation between CIPO patients and controls, with median and IQR indicated in parentheses.

### Assessment of vascular tissue

#### Histochemical orcein staining and small vessel quantitative analysis

Orcein staining was used to label blood vessels and measure the vascular area on  3 FFPE tissue sections (5 µm) from each subject. The stained sections were used to measure the vascular area and to quantify the number of vessels detectable in the jejunal submucosa as previously described [9] and detailed in Supporting Information section. Blood vessels were classified into four categories according to their diameter: >301 µm (large), 300–101 µm (medium), 100–51 µm (small) and <50 µm (very small). To calculate differences in vascular area, number of total vessels or the number of each vessel subtype between CIPO patients and controls, a Mann–Whitney test (non‐parametric) was used for each analysis. Statistical significance was set at *p* < 0.05. Data are reported in the results section as per cent variation between CIPO patients and controls, with median and IQR indicated in parentheses.

#### Protein extraction and quantification

Total proteins were extracted from 0.25 g of each jejunal sample using TPER tissue protein extraction reagent in the presence of protease and phosphatase inhibitor cocktail (Thermo Fisher Scientific). Total protein fractions were quantified with the Pierce Coomassie Plus Protein Assay Reagent (Thermo Fisher Scientific) on a DU730 Life Science UV/VIS spectrophotometer and were stored at −80°C. Data are reported in the results section as per cent variation between CIPO patients and controls, with median and IQR indicated in parentheses.

#### Protein separation and detection using western blotting (WB)

Proteins were separated using 12% acrylamide SDS–PAGE. Samples were diluted (v/v) in Laemmli sample buffer pH 6.8 (4% SDS; 20% glycerol; 120 mmol/L Tris‐Cl; bromophenol blue 0.02%; 10% DTT) and denatured at 95°C for 10 min before loading. Proteins were transferred onto nitrocellulose membrane (Thermo Fisher Scientific) at 350 mA for 1 h at room temperature. Membranes were blocked for 1 h at room temperature with a buffer containing 5% fat‐free milk in phosphate Tris buffer saline (100 mM Tris HCl, 1.5 M NaCl, 0.5% Tween‐20, pH 8.3, all purchased from Merck KGaA) and then incubated for 16 h at 4°C with human monoclonal anti‐TP ab diluted 1:1000 (#MA5‐13542, Invitrogen, Thermo Fisher Scientific), human monoclonal anti‐HIF‐1α antibody diluted 1:500 (#sc‐13515, Santa Cruz Biotechnology) and human monoclonal anti‐VEGF ab diluted 1:500 (#sc‐7269, Santa Cruz Biotechnology). Membranes were washed three times in phosphate‐/Tris‐buffered saline and incubated with anti‐mouse HRP‐conjugated secondary antibody diluted 1:100,000 (Sigma) for 2 h at room temperature. Immunoreactive bands were visualized by ECL western blotting (WB) substrate (Thermo Fisher Scientific) on iBright FL1500 Imaging System (Invitrogen). The iBright software was used to quantitate the total protein amount in each lane, stained with Ponceau. The chemiluminescent signal was captured for each analysed protein and normalized using the total protein normalization factor calculated by the software for the respective ponceau‐stained lane, according to the manufacturer's instructions [23]. For our experiments involving tissue samples, Control 1 was selected as the internal calibrator and was run into all the membranes and assigned as unitarian value. A Mann–Whitney test (non‐parametric) was used to calculate protein expression differences between CIPO and controls. Statistical significance was set at *p* < 0.05.

#### Protein immunolocalization

For all the samples and markers, FFPE sections (5 µm) were deparaffinized in Xylene, rehydrated and antigen retrieval was performed by heating the sections for 25 min at 90°C in a water bath in the presence of 10 mmol/L sodium citrate buffer (pH 6.0). TP and VEGF proteins were detected using immunofluorescence. After 10 min in blocking solution (Millipore), sections were incubated with mouse primary anti‐TP antibody at a final concentration of 0.002 mg/mL (Abcam) or anti‐VEGF antibody at a final concentration of 0.004 mg/mL (#sc‐7269, Santa Cruz Biotechnology) in a humidified chamber overnight at 4°C. The secondary fluorescent antibody (Goat anti‐Mouse IgG (H + L) Highly Cross‐Adsorbed Secondary Antibody, Alexa Fluor Plus 555; dilution 1:10,000) (Thermo Scientific) was incubated for 2 h at room temperature. Fluoroshield DAPI solution (Sigma‐Aldrich) was used as a mounting solution and to counterstain nuclei. HIF‐1α identification was obtained using immunohistochemistry. Sections were treated with an endogenous peroxidase blocking kit (Gene Tex). Immunostaining was performed using a commercial kit (Millipore) following the manufacturer's instructions. Mouse monoclonal anti‐HIF‐1α (sc‐13515; dilution: 0.02 mg/mL) (Santa Cruz Biotechnology) was used to label vessels and tissues and/or cells in hypoxic conditions, respectively. The goat anti‐mouse biotinylated secondary antibodies (dilution 1:1) were used as provided by the immunostaining kit (Millipore). Image capturing was obtained using an Evident/Olympus IX83 inverted microscope coupled with cellsens dimension software 4.3.1.

### mtDNA copy number assessment

The absolute quantification of mtDNA copy number and deletion was performed using droplet digital PCR (BioRad) on DNA extracted, as previously reported [16, 17]. The analysis was carried out on QX200 Droplet Digital PCR System coupled with Quantasoft‐Analysis‐Pro‐1.0 (BioRad). The difference between mtDNA copy number measured in CIPO and controls was assessed using a Mann–Whitney test (non‐parametric). Statistical significance was set at *p* < 0.05. Data are reported in the results section as per cent variation between CIPO patients and controls, with median and IQR indicated in parentheses.

### Molecular and morphological correlations with the number of sub‐occlusive episodes experienced by patients

Multivariate Spearman correlation was applied to assess associations between morphometric and biochemical data and the number of sub‐occlusive episodes (nCIPO), used as an indicator of disease persistence and clinical impact. Specifically, the analysis included the following morphological data: submucosal vascular area (mm^2^); blood vessel density (n^v^/mm^2^ submucosa); the percentage of vessels in each considered dimensional class, that is, large_>301 µm_, medium_300–101 µm_, small_100–51 µm_ and very small_<50 µm_; circular and longitudinal muscle layer thickness; and number of myenteric neurons. The following biochemical and molecular data were also considered: fibrosis index; TP, VEGF and HIF‐1α protein expression levels; and mtDNA copy number. For each comparison, a *p* and an *R* value were generated. A positive *R* indicates a direct correlation, whereas a negative *R* indicates an inverse correlation.

## Results

### Rare and new collagen gene variants are identified in CIPO patients

WES analysis was performed on genomic DNA extracted from patients’ tissue biopsies, and variants were filtered based on frequency in public databases, whereas the effect at the protein level and pathogenicity score were assessed according to a previously published pipeline [24]. The analysis of gene variants associated with known GI/neuromuscular disorders failed to reveal pathogenic or likely pathogenic variants, including in *TYMP*.

Nonetheless, in 15% of cases, we identified three novel or very rare variants in different collagen genes (Table S1) that were predicted as likely pathogenic or variants of uncertain significance (VUS), according to the American College of Medical Genetics and Genomics (Franklin: https://franklin.genoox.com/clinical‐db/home). These variants were never associated to CIPO before. All variants were detected as heterozygous changes. Two likely pathogenic variants were found in two different patients. One in *COL6A1* and one in *COL4A3*, respectively, are associated with dominant forms of Bethlem myopathy (MIM #158810) and Ullrich congenital muscular dystrophy (MIM #254090) and with a dominant form of Alport syndrome (MIM #104200), characterized by severe renal defects. One VUS was identified in *COL10A1* gene, associated with Metaphyseal chondrodysplasia, Schmid type (MIM #156500). None of the herein investigated CIPO patients showed clinical features classically associated with these diseases. Importantly, separate histological and molecular analyses of these patients revealed no significant differences compared to the remaining CIPO cohort in any of the evaluated parameters (data not shown).

### mtDNA haplogroup J is overrepresented in CIPO patients

No mtDNA somatic rearrangements (deletions or duplications), was detected in the jejunum of CIPO patients. All variants identified in the mtDNA sequence analysis (mean coverage 398 ± 140), including the private variants, were classified as benign. The distribution of mtDNA haplogroups in the CIPO cohort differed from that of the controls, with an overrepresentation of haplogroup J (35% in CIPO vs. 8% in controls), thus increasing the risk of developing CIPO (OR = 5.31, raw *p* = 0.0086, adjusted *p* = 0.051) (Table S2).

### CIPO jejunal tissue shows higher fibrosis and longitudinal muscle thinning

**Fig. 1:**
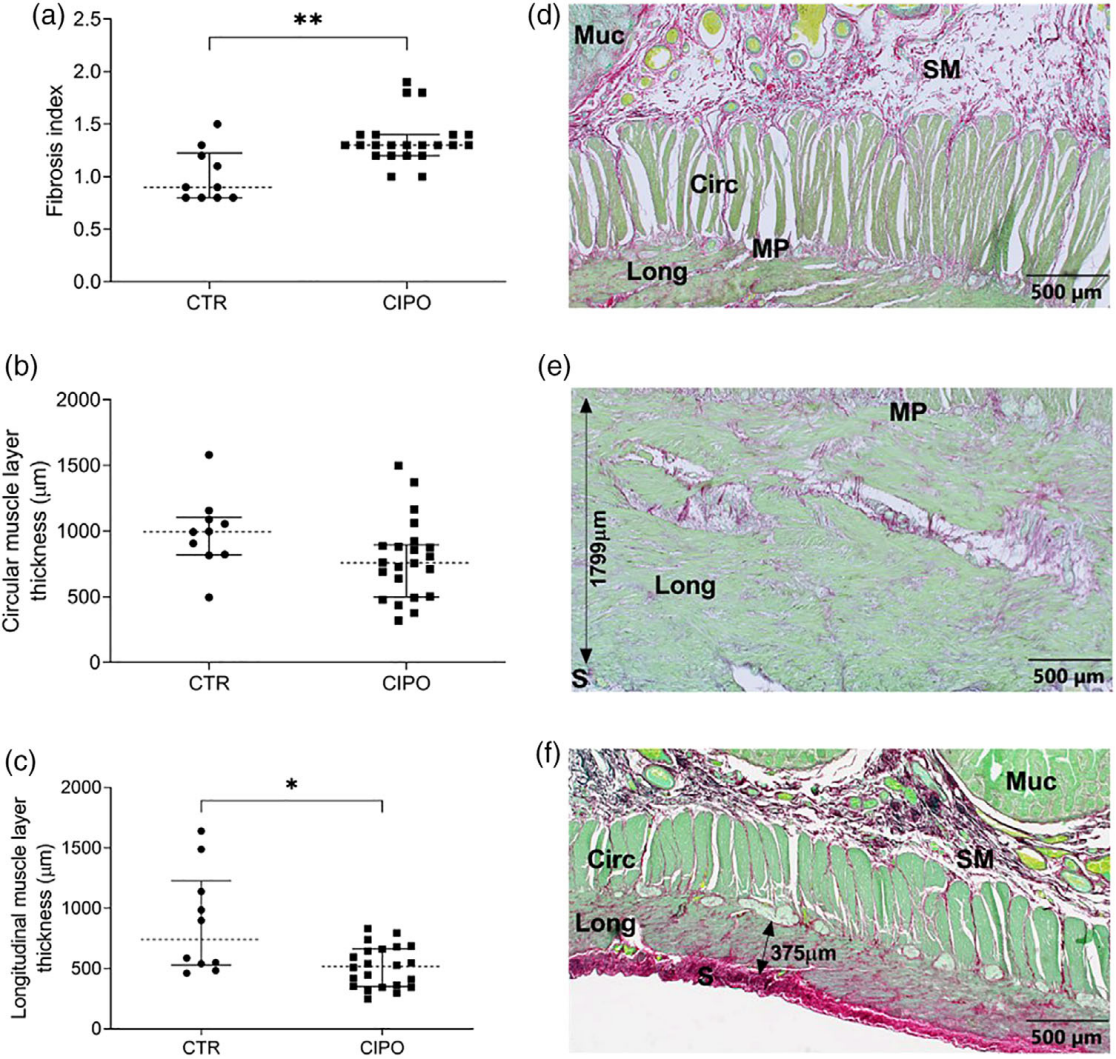
Tissue fibrosis and muscular abnormalities. (a) Tissue fibrosis has been spectrophotometrically calculated and is represented for chronic intestinal pseudo‐obstruction (CIPO) patients versus controls (CTR); **p = 0.0016. Morphometric assessment of muscular components is reported in (b) for the circular muscle layer thickness; and (c) for the longitudinal muscle layer thickness; *p = 0.0121. Sirius Red (collagen—red) and fast green (other non‐collagenous proteins—green) stained sections are reported as follows: (d) one control in which all tissue layers are represented; (e) one control focusing on the longitudinal layer to appreciate its thickness; (f) one section representative of all layers of a CIPO patient. Fibrosis is demonstrated by the enrichment of collagen (red) in all layers. In the picture, fibrosis is observed also in the submucosa. The black arrows in panels (e) and (f) illustrate the thickness of the longitudinal muscle layer. Data in panels (a–c) are presented with median value (dotted line) and interquartile range (25th and 75th percentiles, shown as error bars). Circ, circular muscle layer; Long, longitudinal muscle layer; MP, myenteric plexus; Muc, mucosa; S, serosa; SM, submucosa.

The fibrosis index in CIPO was higher than in controls (CTR), with a median difference of 0.4 [1.3 (IQR 0.2)_CIPO_ vs. 0.9 (IQR 0.4)_CTR_; *p* = 0.0016] (Fig. 1a). The thickness of the circular muscle layer (Fig. 1b) did not vary between CIPO and CTR [994 µm (IQR 304.5)_CTR_ vs. 758.5 µm (IQR 396.8)_CIPO_; *p* = 0.0516 n.s.], whereas the longitudinal (Fig. 1c) muscle layer was significantly thinner, with a median difference of 225.3 µm [743.1 µm (IQR 700.1)_CTR_ vs. 517.8 µm (IQR 314.8)_CIPO_; *p* = 0.0121]. Fig. 1d–f shows collagen accumulation and differences in circular and longitudinal muscle thickness between CTR (Fig. 1d,e) and CIPO (Fig. 1f) samples. Specifically, abundant collagen was detected throughout jejunal layers, predominantly in the circular and/or longitudinal muscle layers, submucosa and around the vascular walls of variously sized vessels. The mucosa was also affected in 30.3% of cases, and a fibrous ‘cap’ encasing myenteric ganglia was observed in approximately 24.2% of CIPO cases.

When calculated in patients subdivided according to the presence (INF) or the absence (NOT‐INF) of an inflammatory infiltrate in the neuromuscular layer, the fibrosis index was higher compared to controls (CTR), with a median difference of 0.4 [1.3 (IQR 0.2)_INF_ vs. 0.9 (IQR 0.4)_CTR_; *p* = 0.0180] and 0.4 [1.3 (IQR 0.3)_NOT_‐_INF_ vs. 0.9 (IQR 0.4)_CTR_; *p* = 0.0035]. No difference was detected between INF and NON‐INF patients (*p* = 0.8438). See Fig. S1.

### CIPO jejunal tissue displays enteric neuronal loss and disrupted myenteric organization

**Fig. 2:**
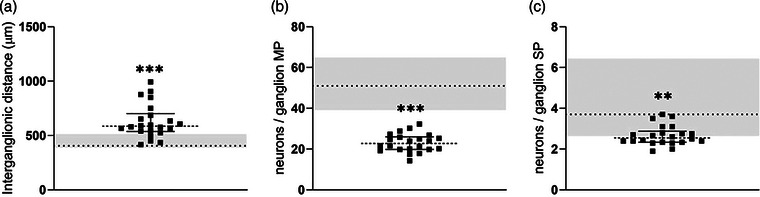
Inter‐ganglionic distance assessment and neuronal cell count. The values measured in chronic intestinal pseudo‐obstruction (CIPO) cohort have been compared to the range of controls CTR) represented by the grey area, where the dotted lines indicate the median value: (a) myenteric inter‐ganglionic distances; ***p = 0.0002; (b) number of myenteric neurons/ganglion; ***p < 0.0001; (c) number of submucosal neurons/ganglion; **p < 0.0012. CIPO data are presented with the median value (dotted line) and interquartile range (25th and 75th percentiles, shown as error bars).

Compared to CTR, the examined CIPO cohort showed a higher inter‐ganglionic distance with a median difference of 180 µm [405 µm (IQR 87)_CTR_ vs. 585 µm (IQR 258.1)_CIPO_; *p* = 0.0002] (Fig. 2a), whereas the number of myenteric neurons/ganglion (Fig. 2b) was lower in CIPO patients compared to CTR with a median difference of 28.2 neurons/ganglion [51.1 (IQR 18.3)_CTR_ vs. 22.8 (IQR 7.3)_CIPO_ neurons/ganglion; *p* < 0.0001]. Submucosal neurons were lower in CIPO as compared to CTR with a median difference of 1.15 neurons/ganglion [3.7 (IQR 2.2)_CTR_ vs. 2.5 (IQR 0.8)_CIPO_ neurons/ganglion; *p* = 0.0012] (Fig. 2c).

### CIPO patients exhibit jejunal microvascular remodelling

**Fig. 3:**
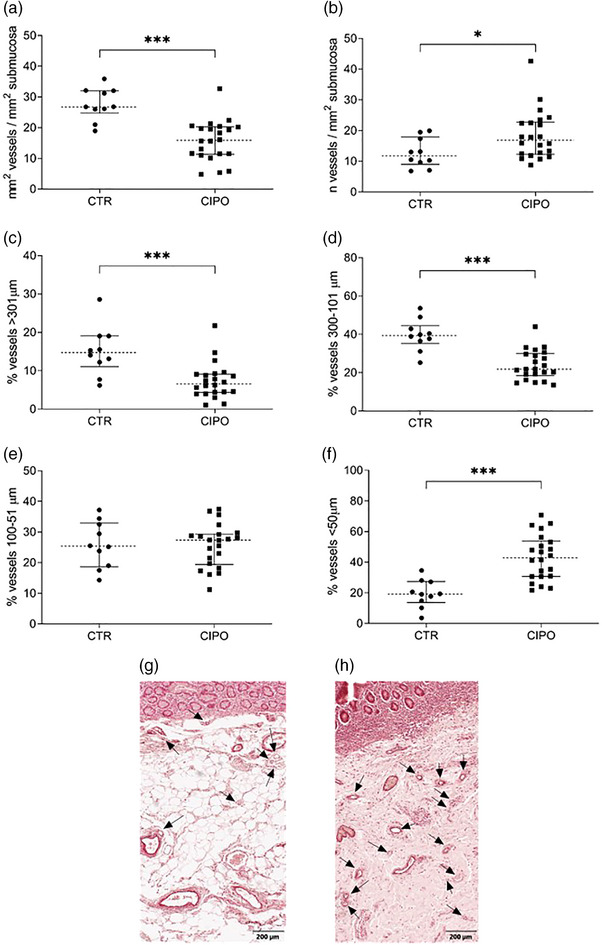
Morphometric assessment of submucosal vascular tissue in chronic intestinal pseudo‐obstruction (CIPO) patients and controls. (a) Ratio of the area of the submucosal layer occupied by vascular tissue, expressed in mm^2^, divided by the total area (mm^2^) of the submucosa; ***p < 0.0001. (b) Ratio of the number of total vessels counted in the submucosa divided by the total area (mm^2^) of the submucosa; *p = 0.0470. The percentage of submucosal vessels with a diameter (c) >301 µm is ***p = 0.0005; (d) between 300 and 101 µm; ***p < 0.0001; (e) between 100 and 51 µm; (f) <50 µm; ***p < 0.0001. Representative images of sections stained with orcein are shown for: (g) a control (CTR) sample and (h) and CIPO sample (magnification bar 200 µm). Arrows indicate vessels <50 µm. Data in panels (a–f) are presented with median value (dotted line) and interquartile range (25th and 75th percentiles, shown as error bars).

Compared to CTR, we detected a lower submucosal vascular area of CIPO patients compared to controls, with a median difference of 10.9 mm^2^ [26.8 mm^2^ (IQR 7.3)_CTR_ vs. 15.9 mm^2^ (IQR 8.7)_CIPO_; *p* < 0.0001] (Fig. 3a), whereas the density of submucosal blood vessels was 30% higher with a median difference of 5.2 n^v^/mm^2^ [11.7 n^v^/mm^2^ (IQR 8.9)_CTR_ vs. 16.9 n^v^/mm^2^ (IQR 10.5)_CIPO_; *p* = 0.0470] (Fig. 3b).

Vessels were also counted and classified according to size (Fig. 3c–f). The percentage of large_>301 µm_ (Fig. 3c) and medium_300–101 µm_ vessels (Fig. 3d) was lower in CIPO compared to CTR, with a median difference of 8.1% and 17.5%, respectively, lower [14.7% (IQR 8.0)_CTRvessels>301 µm_ vs. 6.6% (IQR 4.8)_CIPOvessels>301 µm;_ *p* = 0.0005 and; 39.3% (IQR 9.3)_CTRvessels300–101 µm_ vs. 21.8% (IQR 11.5)_CIPO%vessels300–101 µm_; *p* < 0.0001]. Small_100–51 µm_ vessels (Fig. 3e) did not vary between CIPO and CTR [25.3% (IQR 14.4)_CTRvessels100–51 µm_ vs. 27.3% (IQR 10.0)_CIPOvessels100–51 µm_; *p* = 0.8928 ns]. In contrast, very small_<50 µm_ vessels (Fig. 3f–h) were higher in CIPO compared to CTR with a median difference of 23.8% [19.2% (IQR 13.8)_CTRvessels<50 µm_ vs. 43.0% (IQR 23.3)_CIPOvessels<50 µm_; *p* < 0.0001].

### CIPO jejunal tissue shows reduced TP and VEGF expression

**Fig. 4:**
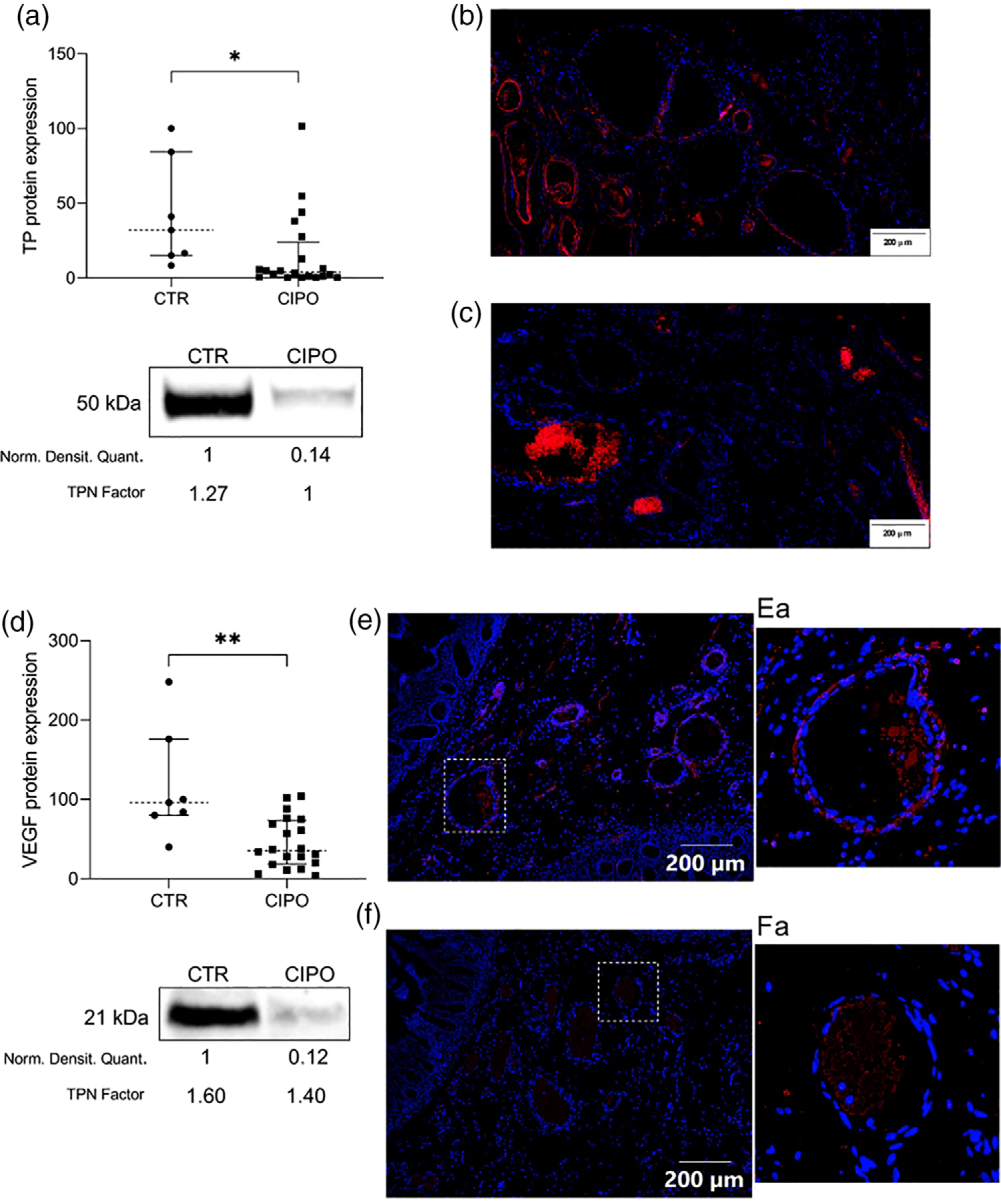
Thymidine phosphorylase (TP) and vascular endothelial growth factor (VEGF) protein level and localization. (a) The jejunal TP enzyme expression is reported with median value (dotted line) and interquartile range (25th and 75th percentiles, shown as error bars) for chronic intestinal pseudo‐obstruction (CIPO) patients and controls (CTR) *p = 0.0112; the picture under the graph displays a cropped example of the detection of TP immunoreactive bands. The normalized densitometric quantification of the immunoreactive bands (Norm. Densitom. Quant.) and the total protein normalization factor (TPN factor) calculated on total proteins are reported below the respective lanes. (b) Immunofluorescence image showing in red the TP‐immunoreactive cells populating the submucosa of the jejunum of a control sample (200 µm magnification bar); (c) TP immunostaining of a CIPO patient (200 µm magnification bar); (d) The jejunal VEGF protein expression is reported with median value (dotted line) and interquartile range (25th and 75th percentiles, shown as error bars) for CIPO patients and CTR; **p = 0.0026; The picture under the graph displays a cropped example of the detection of VEGF immunoreactive bands. The normalized densitometric quantification of the immunoreactive bands (Norm. Densitom. Quant.) and the total protein normalization factor (TPN factor) calculated on total proteins are reported below the respective lanes. (e) Immunofluorescence image showing in red the VEGF‐immunoreactive cells populating the submucosa of the jejunum of a control sample (200 µm magnification bar); and (Ea) a vessel detail as an example of the staining: (f) VEGF immunostaining of a CIPO patient (200 µm magnification bar); and (Fa) a vessel detail as an example of the staining.

Compared to CTR, TP protein expression was lower in CIPO with a median difference of 28.0 AU [32.1 AU (IQR 69.1)_CTR_ vs. 4.1 AU (IQR 23.1)_CIPO_; *p* = 0.0112] (Fig. 4a). We detected TP immunoreactivity (red fluorochrome shown in Fig. 4b) along the control vessel walls, regardless of the vessel size. In CIPO tissues, there was a significant reduction/absence of TP immunoreactivity (Fig. 4c). Compared to CTR, VEGF protein expression was lower in CIPO with a median difference of 60.7 AU [96.0 AU (IQR 96.0)_CTR_ vs. 35.3 AU (IQR 55.0)_CIPO_; *p* = 0.0026] (Fig. 4d), and VEGF immunoreactivity, which was detectable at the edge of the vessel wall of CTR tissue (Fig. 4e‐Ea), was conversely fragmented/absent in CIPO vessels (Fig. 4f‐Fa).

### Tissue hypoxia is detected in CIPO jejunal tissue, despite preserved mtDNA copy number

**Fig. 5:**
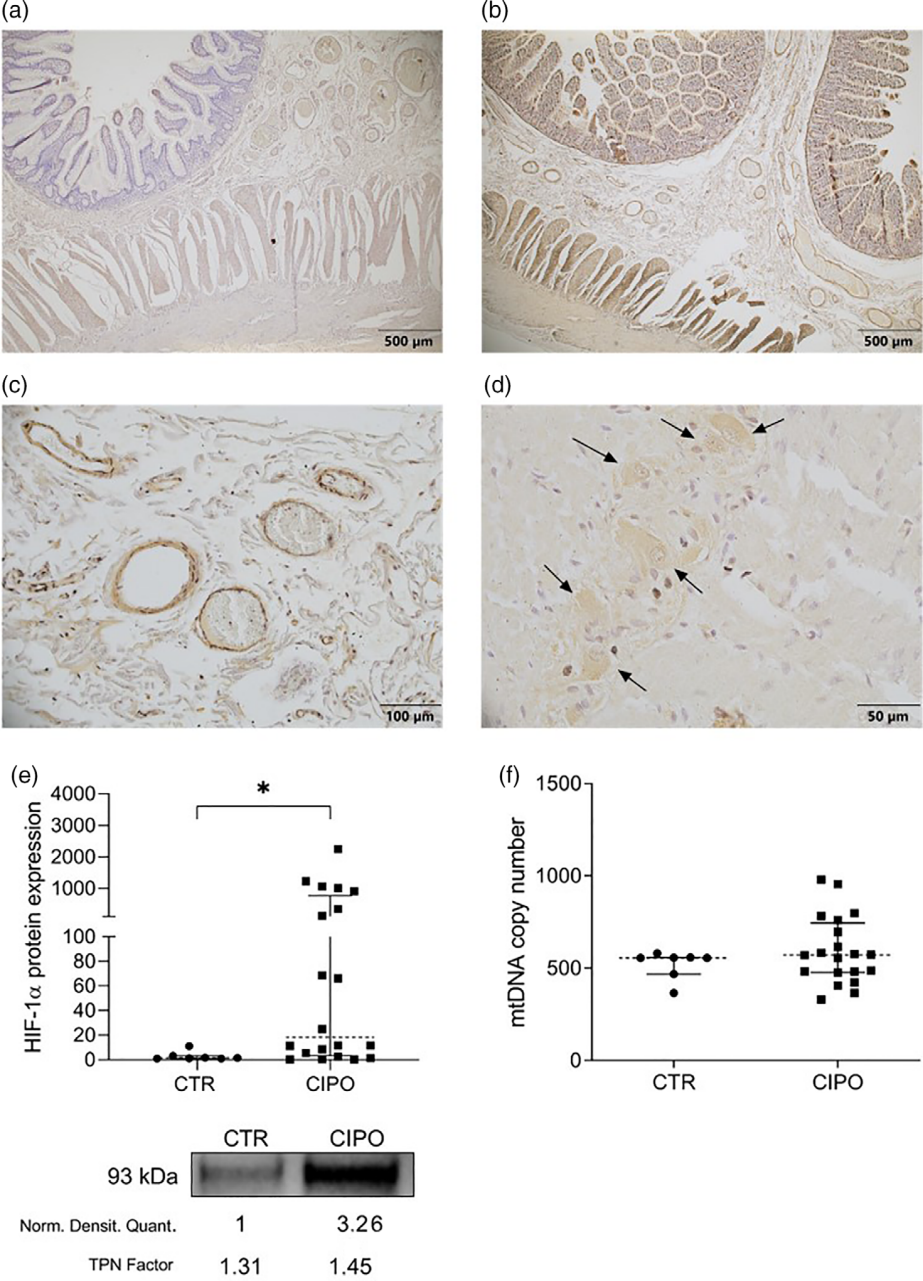
Hypoxia inducible factor‐1α (HIF‐1α) localization, HIF‐1α expression and mtDNA assessment. HIF‐1α immunolocalization (500 µm scale bar) in representative control (CTR) (a) and chronic intestinal pseudo‐obstruction (CIPO) patient (b). Higher magnification of a CIPO sample for (c) small vessels belonging to the submucosal layer (100 µm scalebar); and (d) black arrows indicate neurons of the myenteric plexus expressing HIF‐1α (50 µm scalebar). (e) Jejunal HIF‐1α expression measured using western blotting (WB) *p = 0.0162. The picture under the graph reports a cropped example of the detection of HIF‐1α immunoreactive bands; the normalized densitometric quantification of the immunoreactive bends (Norm. Densitom. Quant.) and the total protein normalization factor (TPN factor) calculated on total proteins are reported below the respective lanes. (f) The graph shows the number of mtDNA copies. Data in panels (e and f) are presented with median value (dotted line) and interquartile range (25th and 75th percentiles, shown as error bars).

HIF‐1α, a marker of tissue hypoxia, was localized using immunohistochemistry (Fig. 5a–d) and quantified with WB (Fig. 5e). The quantification of HIF‐1α protein expression revealed that this marker was higher in CIPO compared to CTR with a median difference of 18.1 AU [1.5 AU (IQR 2.1)_CTR_ vs. 18.2 AU (IQR 767.5)_CIPO_; *p* = 0.0162]. HIF‐1α immunoreactivity was visualized in the jejunal epithelium, vascular wall and submucosal and myenteric neurons in CIPO specimens. On the other hand, the bulk mtDNA copy number from jejunal tissue did not vary between CIPO and CTR [556.0 copies (IQR 152.5)_CTR_ vs. 572.0 copies (IQR 267.5)_CIPO_; *p* = 0.3412 n.s.] (Fig. 5f).

### Multivariate analysis links CIPO jejunal tissue alterations to clinical severity

The *p* and *R* values concerning the multivariate correlation between biochemical, structural parameters and nCIPO were summarized in Table 1. A lower number of large_>301 µm_ vessels and a smaller vascularized area of the submucosa, along with a higher number of very small_<50 µm_ vessels, correlated with higher fibrosis. A smaller vascular area was also associated with a rewiring of vascular stoichiometry characterized by a lower number of large_>301 µm_ and medium‐sized_300–101 µm_ vessels and a higher number of very small_<50 µm_ ones. It also correlated with thinning of the longitudinal muscle layer, a lower number of myenteric neurons and a lower VEGF protein expression. A lower number of large_>301 µm_ and medium‐sized_300–101 µm_ vessels correlated with a lower number of myenteric neurons and with a higher inter‐ganglionic distance. A higher number of very small vessels_<50µm_ also correlated with a reduced number of myenteric neurons. The downregulation of TP expression correlated with VEGF downregulation and with a higher HIF‐1α expression. Finally, a high frequency of CIPO episodes was associated with a reduced vascular area, a lower number of large_>301 µm_ and medium‐sized_300–101 µm_ vessels, a higher number of very small_<50 µm_ vessels and thinning of the longitudinal muscle layer with lower VEGF expression.

**Table 1:** Multivariate correlations analysis results.

Biochemical/Structural parameter	Biochemical/Structural parameter	*p* value	*R* value
Fibrosis	Number of large vessels	0.002	−0.587
	Number of very small vessels	0.010	0.514
	Vascular area	0.032	−0.448
Vascular area	Number of large vessels	<0.0001	0.677
	Number of medium vessels	0.002	0.515
	Number of very small vessels	<0.0001	−0.551
	Longitudinal muscle layer thickness	0.020	0.422
	Number of myenteric neurons	0.044	0.467
	VEGF expression	0.025	0.499
Number of large vessels	Myenteric inter‐ganglionic distance	0.030	0.484
	Number of myenteric neurons	0.020	−0.530
Number of medium vessels	Myenteric inter‐ganglionic distance	0.017	0.526
	Number of myenteric neurons	0.039	−0.477
Number of very small vessels	Number of myenteric neurons	0.017	−0.526
TP expression	VEGF expression	0.020	0.452
	HIF1‐α expression	0.043	−0.408

Clinical manifestation	Biochemical/Structural parameter	*p* value	*R* value
Number of sub‐occlusive episodes	VEGF expression	0.013	−0.470
	Vascular area	0.010	−0.450
	Number of large vessels	0.023	−0.393
	Number of medium vessels	0.003	−0.502
	Number of very small vessels	0.004	0.485
	Longitudinal muscle layer thickness	0.021	−0.405

*Note*: *p* and *R* values of the calculated correlations are reported. A Negative *R* indicates an inverse relationship between the two examined parameters. Conversely, a Positive *R* Indicates a direct relationship. *p* values were calculated using spearman's non‐parametric correlation test.

## Discussion

Characterizing the mechanisms underlying CIPO remains a major challenge in neurogastroenterology. Our study emphasizes the role of enteric microvasculature and collagen remodelling as central contributors to CIPO. By integrating genetic, morphometric and molecular data, we provided evidence supporting the involvement of previously unexplored pathophysiological mechanisms.

Given the clinical heterogeneity of CIPO and the absence of a consistent pathological signature, we first performed WES to investigate possible genetic contributors in our cohort. Indeed, genetic causes are more commonly detected in syndromic or paediatric forms of CIPO [8]. WES analysis did not identify pathogenic variants in known CIPO‐associated genes. A small proportion of patients (15%) exhibited rare or novel variants in collagen‐related genes, none of which have previously been associated with CIPO. Although these findings suggest a possible collagen‐related genetic susceptibility in a subset of patients, their clinical relevance remains to be clarified and warrants further investigation.

Expanding the genetic investigation on mtDNA, we did not identify any abnormalities in mtDNA sequence. However, 28% of the CIPO patients in our cohort carried mtDNA haplogroup J. Mitochondrial haplogroups are shaped by evolutionary pressures, such as climate, diet or pathogen exposure, and have been linked to increased disease susceptibility [25]. In particular, haplogroup J has been associated with neurological and mitochondrial disorders, as well as with heightened sensitivity to environmental exposures [26, 27, 28]. Within this framework, the enrichment of haplogroup J in our cohort, although not statistically significant, may represent a biologically plausible signal, suggesting that this mitochondrial background could influence individual susceptibility or cellular responses rather than act as a primary pathogenic driver. To our knowledge, this is the first time that haplogroup J has been associated with GI motility disorders.

Although the genetic contributors identified in our cohort appeared limited, the presence of rare collagen‐related variants was particularly intriguing given their potential link to structural alterations. This prompted us to assess collagen organization directly in jejunal tissue to determine whether patients carrying these variants displayed distinct features; unexpectedly, our analysis revealed a higher collagen content throughout the jejunal wall in all CIPO patients. Fibrosis was evident in the submucosa, within both the longitudinal and circular muscle layers and around the myenteric plexus. Remarkably, collagen accumulation has been reported in CIPO‐MNGIE [9] and *LIG3* (a MNGIE‐like syndrome) mutations [29]. Another notable feature was the thinning of the longitudinal muscle layer, whereas the circular layer appeared relatively preserved. These structural changes were accompanied by a 50% reduction in enteric neuron density and a higher inter‐ganglionic distance, consistent with previous observations [6]. Similar neuromuscular alterations have also been described in CIPO‐MNGIE [9]. These findings are particularly relevant given the essential roles of intestinal smooth muscle and enteric neurons in coordinating peristalsis, which is markedly impaired in CIPO patients.

Collagen is a critical component of the extracellular matrix and plays an essential role in maintaining vascular integrity. Abnormalities or disorganization of extracellular matrix components, including collagen, can compromise the stability of the basement membrane, resulting in altered vascular architecture [30]. Given the observed collagen abnormalities, we conducted a detailed morphometric analysis of jejunal submucosal vessels. Our analysis revealed a significant reduction in vascular area, a higher proportion of very small_<50 µm_ vessels and a lower number of medium_300‐101 µm_ and large_>301 µm_ vessels. Remarkably, these findings resembled those previously described in CIPO‐MNGIE [9] and *LIG3*‐related CIPO [29], supporting the hypothesis that vascular remodelling is a pathological feature across various CIPO subtypes. Overall, our findings suggest that idiopathic and genetic forms (e.g., MNGIE) of CIPO may converge on the same key pathogenic pathways, particularly those involving vascular abnormalities and extracellular matrix remodelling. This convergence likely explains why both forms exhibit a similar combination of hypoxia, fibrosis, longitudinal muscle thinning, vascular remodelling and marked neuronal loss, ultimately leading to an overlapping clinical phenotype. Additionally, since fibrosis might result from an underlying inflammatory process [31, 32, 33], we stratified our cohort into 8 ‘inflamed’ patients (6 with mast‐cell infiltrates in the neuromuscular layer, and  2 showing both mast cells and lymphocytes) versus 14 ‘not‐inflamed’ patients. These analyses did not reveal any meaningful differences in structural or molecular parameters between the two subgroups. Although these numbers do not allow definitive conclusions, our data suggest that inflammation is unlikely to represent a major driver of fibrosis in this cohort.

Based on these structural similarities with CIPO‐MNGIE, we hypothesized that similar molecular mechanisms may occur in different forms of CIPO, with CIPO‐MNGIE being the ‘tip‐of‐the‐iceberg’ of GI dysmotility because of its clinical severity and virtually unavoidable complications. In CIPO‐MNGIE, the genetically driven deficiency of TP leads to nucleoside accumulation, mitochondrial dysfunction and vascular abnormalities [10]. Notably, our group observed that MNGIE patients who received liver transplantation as an exogenous source of TP did not recover TP levels in the GI tract, despite systemic metabolic correction [34]. The failure to restore local TP expression appears to affect vascular repair or remodelling in the gut, as TP needs to be expressed in situ to initiate the chemoattraction of endothelial cells necessary for vessel formation or repair [35]. Based on these findings, we decided to evaluate TP expression specifically within the GI tract. Although MNGIE diagnosis was excluded in our cohort through enzymatic and genetic tests, we observed a significant, localized reduction in TP protein within the intestinal tissue of the CIPO patients included in this study. TP, also referred to as platelet‐derived endothelial cell growth factor [36], plays a central role in promoting endothelial cell migration and angiogenesis. Therefore, its local downregulation might impair vascular regeneration in the gut, even in the absence of systemic metabolic abnormalities. These findings on TP further support the possibility that the vascular component plays an important role in CIPO pathogenesis. Hence, we expanded our analysis on VEGF, another critical angiogenic factor involved in vascular remodelling and repair, whose expression is influenced by TP levels [37]. In MNGIE, VEGF expression is upregulated as a plausible compensatory response to TP deficiency [9]. In contrast, VEGF resulted in downregulated in CIPO patients, suggesting that no compensatory effect [9]. The absence of this compensatory mechanism may contribute to an impaired vascular remodelling and microvascular abnormalities.

Altogether, these findings indicated a localized downregulation of angiogenic signalling in the GI tissues of CIPO patients. Although the precise upstream triggers remain to be elucidated, the reduced expression of both TP and VEGF can explain, at least in part, the vascular alterations we demonstrated in this study, including reduced vessel area and altered vessel size. This finding provided the rationale to examine GI tissue oxygenation via the expression of HIF‐1α. We identified a generalized hypoxic state, evidenced by a widespread HIF‐1α expression. This marker was particularly localized around submucosal blood vessels and in some neurons of the myenteric plexus. This hypoxic pattern closely resembles what we observed in CIPO‐MNGIE tissue [9], further suggesting the activation of common pathogenic mechanisms.

Given the observed downregulation of TP and the presence of diffuse tissue hypoxia, we also investigated whether mtDNA copy number was affected in the jejunum of CIPO patients. This analysis was prompted by the well‐established link between TP deficiency and mtDNA depletion. In MNGIE, systemic TP loss impairs nucleoside metabolism, resulting in reduced mtDNA content and mitochondrial dysfunction [11, 34, 38, 39]. Furthermore, hypoxia is known to disrupt mitochondrial biogenesis and mtDNA replication [40]. Despite these converging stressors, the quantification of mtDNA copy number in the jejunal tissue of CIPO patients did not reveal significant differences compared to controls. This finding suggested that, although TP is locally downregulated and tissue hypoxia is present, systemic nucleoside clearance remains intact, thereby preventing secondary mtDNA depletion. In contrast to MNGIE, where TP deficiency is systemic and leads to widespread mitochondrial damage [10], the localized nature of TP loss in CIPO does not appear sufficient to alter mtDNA copy number.

Finally, to better understand how all these biochemical and structural alterations relate to disease manifestation, we conducted a multivariate correlation analysis. Our results demonstrated that reduced vascular area and higher fibrosis were associated with lower TP and VEGF expression, greater expression of HIF‐1α and a higher frequency of sub‐occlusive episodes. Although the underlying mechanisms responsible for the downregulation of TP and other molecular alterations remain to be elucidated and warrant further investigation, our data are consistent with a mechanistic model in which local TP deficiency may lead to impaired angiogenic signalling, thereby contributing to tissue hypoxia, fibrotic remodelling and ultimately neuromuscular degeneration.

## Conclusion

This study provides novel insights into the pathophysiological mechanisms underlying CIPO, one of the most severe clinical phenotypes of gut dysmotility with recurrent sub‐obstructive crisis. Through an integrated approach combining genetic screening, morphometric analyses and molecular profiling, we demonstrated that collagen accumulation, microvascular remodelling, neuromuscular degeneration and angiogenic dysregulation are features detectable in CIPO. WES and mtDNA analyses, respectively, excluded known pathogenic variants and mtDNA rearrangements while highlighting rare collagen gene variants and an overrepresentation of mitochondrial haplogroup J. Taken together, these findings reveal coherent biological signals that open promising avenues for further investigation. Morphometric assessments revealed widespread fibrosis, a significant thinning of the longitudinal muscle layer and marked neuronal loss in both myenteric and submucosal ganglia, with higher inter‐ganglionic distance. These structural abnormalities were accompanied by a profound remodelling of the submucosal vasculature, characterized by a reduced vascular area, depletion of large and medium‐sized vessels and a compensatory increase in very small vessels. Molecular analyses showed a significant downregulation of the angiogenic mediators TP and VEGF, with parallel upregulation of HIF‐1α, indicating a hypoxic tissue environment. Importantly, despite the hypoxic state, mtDNA copy number remained preserved, distinguishing idiopathic CIPO from mitochondrial forms, for example, MNGIE. Finally, the multivariate correlation analysis provided a link between bench and bedside, demonstrating that vascular abnormalities, loss of TP/VEGF expression, tissue hypoxia and muscular and neuronal loss all significantly correlate with the frequency of sub‐occlusive episodes, a clinical hallmark of CIPO. Together, these results support a model in which collagen remodelling, microvascular abnormalities and impaired angiogenesis contribute to neuromuscular alterations underlying CIPO. The correlation of these structural and molecular abnormalities with the frequency of sub‐occlusive episodes reinforces their clinical relevance. Future investigations aimed at validating vascular biomarkers as predictors of disease severity and exploring angiogenesis‐targeted strategies as potential therapeutic avenues in CIPO are eagerly warranted.

## Conflict of interest statement

The authors declare no conflicts of interest.

## Funding information

This work was supported by the European Union #NEXTGENERATIONEU (NGEU) and funded to Prof. Stefano Ratti and Prof. Valerio Carelli by the Italian Ministry of University and Research (MUR), National Recovery and Resilience Plan (PNRR), project MNESYS (PE0000006)—A Multiscale integrated approach to the study of the nervous system in health and disease (DN. 1553 11.10.2022). The work was also supported by Alma Attrezzature 2022 of the University of Bologna funded to Prof. Stefano Ratti, by Telethon grants GGP15171 and GMR23T1033, and by the Italian Ministry of University and Research (MUR) grant PRIN 2022 number 2022A5AB2S_002 funded to Prof. Roberto De Giorgio and Prof. Elena Bonora.

## Patient consent statement

All patients provided written informed consent to participate in the study, which was conducted in accordance with the Declaration of Helsinki.

## Supporting information




**Supporting Table 1**: joim70078‐sup‐0001‐TableS1.xlsx.

**Supporting Table 2**: joim70078‐sup‐0002‐TableS2.pptx.

**Supporting Fig. 3**: joim70078‐sup‐0003‐FigureS1.pdf.

**Supporting File 1**: joim70078‐sup‐0004‐SubMat.docx.

## Data Availability

Data, analytic methods and study materials will be made available to other researchers upon request to our laboratory.
