# Supplementary material for: Collagen and microvascular alterations contribute to neuromuscular degeneration and disease progression in chronic intestinal pseudo‐obstruction
Source: J Intern Med. 2026 Feb 27;299(5):587–603. doi: 10.1111/joim.70078 (PMC13061101; doi:10.1111/joim.70078)
Supplement: Supplementary file 2 — Supporting Table 2: joim70078‐sup‐0002‐TableS2.pptx. [file JOIM-299-587-s003.pptx]

## Slide 1
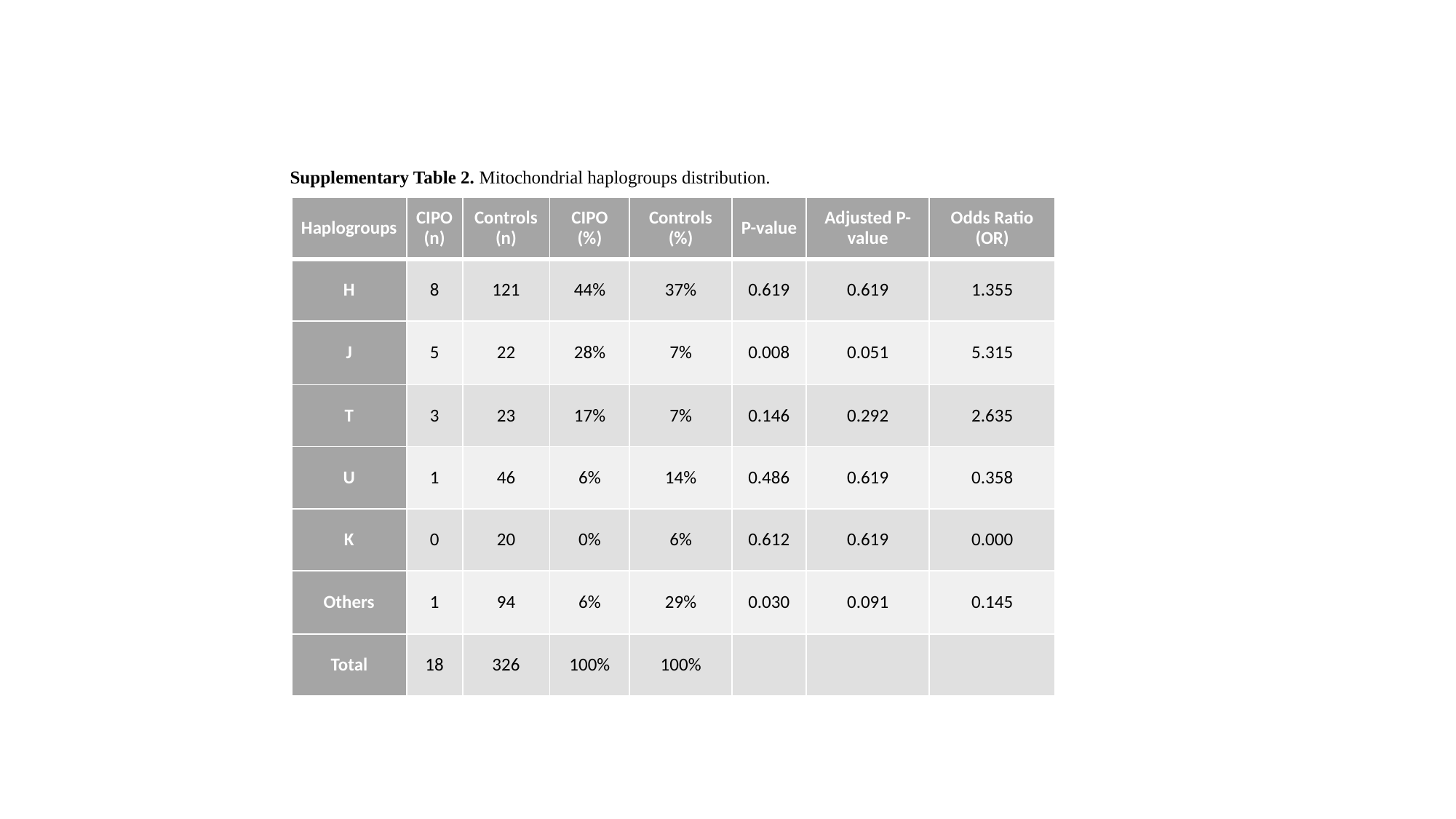

Supplementary Table 2. Mitochondrial haplogroups distribution.
| Haplogroups | CIPO (n) | Controls (n) | CIPO (%) | Controls (%) | P-value | Adjusted P-value | Odds Ratio (OR) |
| --- | --- | --- | --- | --- | --- | --- | --- |
| H | 8 | 121 | 44% | 37% | 0.619 | 0.619 | 1.355 |
| J | 5 | 22 | 28% | 7% | 0.008 | 0.051 | 5.315 |
| T | 3 | 23 | 17% | 7% | 0.146 | 0.292 | 2.635 |
| U | 1 | 46 | 6% | 14% | 0.486 | 0.619 | 0.358 |
| K | 0 | 20 | 0% | 6% | 0.612 | 0.619 | 0.000 |
| Others | 1 | 94 | 6% | 29% | 0.030 | 0.091 | 0.145 |
| Total | 18 | 326 | 100% | 100% | | | |
