# Supplementary figures and images for: Collagen and microvascular alterations contribute to neuromuscular degeneration and disease progression in chronic intestinal pseudo‐obstruction
Source: J Intern Med. 2026 Feb 27;299(5):587–603. doi: 10.1111/joim.70078 (PMC13061101; doi:10.1111/joim.70078)

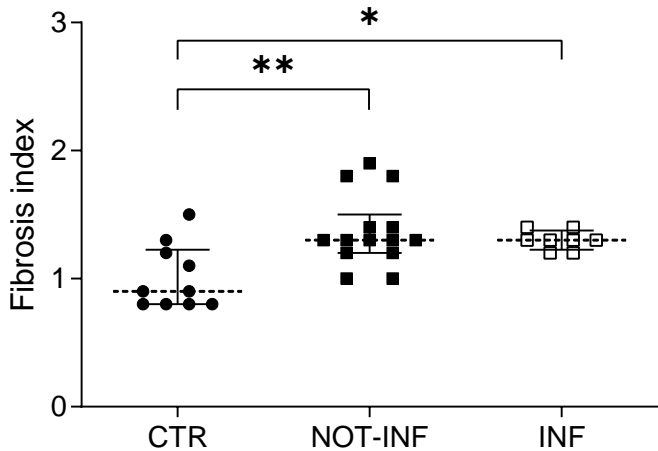

Supplement: Supplementary file 3 — Supporting Fig. 3: joim70078‐sup‐0003‐FigureS1.pdf. [file JOIM-299-587-s004.pdf]
