# Supplementary material for: Collagen and microvascular alterations contribute to neuromuscular degeneration and disease progression in chronic intestinal pseudo‐obstruction
Source: J Intern Med. 2026 Feb 27;299(5):587–603. doi: 10.1111/joim.70078 (PMC13061101; doi:10.1111/joim.70078)
Supplement: Supplementary file 4 — Supporting File 1: joim70078‐sup‐0004‐SubMat.docx. [file JOIM-299-587-s002.docx]

**SUPPLEMENTARY MATERIALS**

**Clinical diagnosis information**

The clinical diagnosis of CIPO was established on the basis of chronic (>3 months) severe symptoms mimicking mechanical small bowel obstruction, and (at least on one occasion) radiological evidence of air-fluid levels and/or dilated small bowel loops. Abdominal distension was reported in 100% of patients, while 86.4% experienced severe and abdominal pain. These symptoms were associated with nausea in 36.4% of cases, vomiting in 27.3%, and radiological evidence of chronic small bowel dilatation in 27.3%. Malnutrition, based on age, sex, and BMI, was present in 59.1% of patients. Parenteral nutrition was necessary in 27.3% of cases, and among these, 66.7% experienced central venous catheter related infections. A sensation of fullness and early satiety was reported in 45.5% of patients. Altered bowel habits were also observed: 40.9% had constipation, whereas 31.8% had diarrhoea. More diffuse gastrointestinal symptoms were reported, including oesophageal involvement in 13.6% of cases, gastroparesis in 27.3%, and urinary symptoms in 9.1%. Small intestinal bacterial overgrowth was documented by duodenal juice cloture in 54.5% of patients. Mechanical obstruction had been ruled out through standard imaging techniques or previous exploratory abdominal surgery.

**Myenteric and submucosal neuronal quantification and myenteric inter-ganglionic distance assessment**

Neurons were stained in three sections per sample (controls and CIPO) via immunohistochemistry using the pan-neuronal marker neuron-specific enolase (NSE; rabbit PA1-28217, ready-to-use antibody; Thermo Fisher Scientific, Rockford, USA). The distance between myenteric ganglia was assessed in adjacent microscopic fields from randomly selected sections. All consecutive fields were analysed, starting from the first detectable ganglion at one extremity of a given section and proceeding toward the opposite end. At least three randomly selected, non-consecutive jejunal cross-sections from one specimen per patient and control were examined. Since the exact boundaries of each ganglion in FFPE sections cannot be precisely defined, we considered clusters of NSE-immunoreactive neuronal cells located within 300 µm of each other as a single arbitrary ganglionic unit. Once defined each arbitrary ganglionic unit, the inter-ganglionic distance and the neuronal cells counted was performed by two investigators (blinded with regard to CIPO or control origin).

**Histochemical orcein staining and small vessels quantitative analysis**

n=3 FFPE tissue sections (5µm) from each subject were deparaffinized in xylene and rehydrated through graded ethanol. After 1 hour of incubation with a solution containing 1% orcein, 70% ethanol and 0.6 cc HCl (ethanol, HCl, orcein powder and xylene were purchased from Carlo Erba, Milan, Italy), samples were washed 5 seconds in distilled water and 30 seconds with 95% ethanol. Finally, the sections were kept for 30 minutes in a 100% ethanol solution to allow the elastic fibers to assume the typical black-brown color. To remove staining from non-elastic fibers, sections were treated with a solution containing 70% ethanol and 1%HCl for 5 minutes. After dehydration, slides were cover-slipped using DPX mounting medium (Sigma-Aldrich, Milan, Italy).

Orcein-stained sections were used to measure the vascular area (i.e., the proportion of tissue occupied by blood vessels within the submucosa) and to quantify the number of vessels detectable in the jejunal submucosa. In each section, all consecutive fields representative of the submucosal layer (at least 10 fields per section) were acquired using a Nikon DXM1200 digital camera (Nikon, Tokyo, Japan) mounted on a LEICA DM LB light transmission microscope (Leica, Mannheim, Germany) at 100× final magnification. Images were analyzed using the Automatic Camera Tamer (ACT)-1 software (Nikon, Tokyo, Japan). For each control and patient section, the total vascular area was calculated relative to the total submucosal area (expressed as a ratio). Vessel density was expressed as the number of vessels per mm² of submucosa. The diameter of each vessel was determined using ImageJ 1.48V software, consistently considering the largest axis in asymmetrical profiles. Blood vessels were classified into four categories according to their diameter: >301 µm (large), 300-101 µm (medium), 100–51 µm (small), and <50 µm (very small). For each subject, the proportion of vessels within each size category was expressed as a percentage of the total vessel count. All analyses were independently performed in a blinded manner by two investigators, with both operators remaining unaware of the experimental group assignment throughout the evaluation.

**Supplementary Figure 1 Legend Tissue Fibrosis in patients with or without a histochemically detected neuromuscular inflammation.** Tissue fibrosis was calculated spectrophotometrically and is shown for CIPO inflamed patients (INF) versus CIPO non-inflamed patients (NOT-INF) and versus controls (CTR). CTR vs. NOT-INF **P=0.0035; CTR vs. INF *P=0.0180 Data are presented with median value (dotted line) and interquartile range (25^th^ and 75^th^ percentiles, shown as error bars).

**Supplementary Table 1 Legend.** Demographic, clinical, and genetic features of the investigated CIPO cohort at the time of jejunal biopsy. The table reports, for each patient, age, age at symptom onset, disease duration, sex, body max index (BMI), need for central venous catheter (CVC) and related infections, number of sub-occlusive (CIPO) episodes prior to biopsy, major gastrointestinal and extra-gastrointestinal symptoms, evidence of chronic small bowel dilatation, and availability of frozen tissue. For patients carrying variants identified through whole-exome sequencing (WES), the corresponding chromosomal location, genomic position and affected gene, Franklin pathogenicity classification, associated Online Mendelian Inheritance in Man (OMIM) disease (when applicable), database of Single Nucleotide Polymorphism (dbSNP) reference, minor allele frequency (MAF), and Combined Annotation Dependent Depletion (CADD) score are provided.

**Notes to Supplementary Table 1**. Although all patients experienced at least one radiologically confirmed pseudo-obstructive episode with air-fluid levels, only a minority showed chronic small-bowel dilatation at the time of biopsy. This is consistent with previous series reporting that persistent dilatation is not uniformly present in CIPO and may reflect a dynamic feature influenced by intestinal tone and contractility, and that chronic or persistent dilatation may represent an advanced stage of disease [1]. Importantly, jejunal biopsies were obtained at a specific time point in each patient’s clinical course, which does not necessarily correspond to the cumulative disease duration nor capture the episodic nature of motility impairment. In line with this, the proportion of patients requiring parenteral nutrition (PN) reflects only those who were dependent on prolonged PN at the time of biopsy. Because nutritional support in CIPO often fluctuates according to disease severity and episodic deterioration, historical or transient PN requirements were not included. This explains the relatively low percentage observed in our cohort and is consistent with previous series showing that long-term PN is needed only in a subset of CIPO patients with persistent or severe motility impairment [2, 3].

**References**

1 Stanghellini V, Camilleri M, Malagelada JR. Chronic idiopathic intestinal pseudo-obstruction: clinical and intestinal manometric findings. *Gut* 1987; **28:** 5-12.

2 Joly F, Amiot A, Messing B. Nutritional support in the severely compromised motility patient: when and how? *Gastroenterol Clin North Am* 2011; **40:** 845-51.

3 Kirby DF, Raheem SA, Corrigan ML. Nutritional Interventions in Chronic Intestinal Pseudoobstruction. *Gastroenterol Clin North Am* 2018; **47:** 209-18.
